# Association of the Aspartate Aminotransferase to Alanine Aminotransferase Ratio with BNP Level and Cardiovascular Mortality in the General Population: The Yamagata Study 10-Year Follow-Up

**DOI:** 10.1155/2016/4857917

**Published:** 2016-10-31

**Authors:** Miyuki Yokoyama, Tetsu Watanabe, Yoichiro Otaki, Hiroki Takahashi, Takanori Arimoto, Tetsuro Shishido, Takuya Miyamoto, Tsuneo Konta, Yoko Shibata, Makoto Daimon, Yoshiyuki Ueno, Takeo Kato, Takamasa Kayama, Isao Kubota

**Affiliations:** ^1^Department of Cardiology, Pulmonology, and Nephrology, Yamagata University School of Medicine, Yamagata, Japan; ^2^Department of Endocrinology and Metabolism, Hirosaki University Graduate School of Medicine, Aomori, Japan; ^3^Global Center of Excellence Program Study Group, Yamagata University School of Medicine, Yamagata, Japan

## Abstract

*Background*. Early identification of high risk subjects for cardiovascular disease in health check-up is still unmet medical need. Cardiovascular disease is characterized by the superior increase in aspartate aminotransferase (AST) to alanine aminotransferase (ALT). However, the association of AST/ALT ratio with brain natriuretic peptide (BNP) levels and cardiovascular mortality remains unclear in the general population.* Methods and Results*. This longitudinal cohort study included 3,494 Japanese subjects who participated in a community-based health check-up, with a 10-year follow-up. The AST/ALT ratio increased with increasing BNP levels. And multivariate logistic analysis showed that the AST/ALT ratio was significantly associated with a high BNP (≥100 pg/mL). There were 250 all-cause deaths including 79 cardiovascular deaths. Multivariate Cox proportional hazard regression analysis revealed that a high AST/ALT ratio (>90 percentile) was an independent predictor of all-cause and cardiovascular mortality after adjustment for confounding factors. Kaplan-Meier analysis demonstrated that cardiovascular mortality was higher in subjects with a high AST/ALT ratio than in those without.* Conclusions*. The AST/ALT ratio was associated with an increase in BNP and was predictive of cardiovascular mortality in a general population. Measuring the AST/ALT ratio during routine health check-ups may be a simple and cost-effective marker for cardiovascular mortality.

## 1. Introduction

Despite advances in medicine, cardiovascular disease is still a public health problem with an increasing prevalence and high mortality [1]. It is becoming increasingly evident that it would be beneficial to identify high risk subjects for future cardiovascular disease during routine health check-ups.

Brain natriuretic peptide (BNP), which is secreted from the left ventricle through mechanical stretch, is a gold standard biomarker for the diagnosis and prognosis of heart failure [2]. Increased BNP levels are reported to be associated with all-cause and cardiovascular mortality in the general population, even in the case of a slight increase in BNP [3, 4]. Although high BNP is useful for early identification of high risk subjects for cardiovascular disease, it is not routinely measured in health check-ups in general population, suggesting the importance of the surrogate marker, which is routinely measured in health check-ups.

Cardiohepatic interaction has been noted in patients with heart failure [5], atrial fibrillation [6], and myocardial infarction [7]. Although the relationship between aminotransferase levels and cardiovascular disease has become a matter of discussion [8, 9], the prognostic utility of aminotransferase has not been fully studied.

Aminotransferase is a well-known marker for liver injury and is composed of alanine aminotransferase (ALT) and aspartate aminotransferase (AST). ALT is only located in the liver, but AST is located in both the liver and myocardial tissue. Cardiac disease leads to hypoxic hepatitis, which results in a rise in serum transaminase activity caused by anoxic necrosis of the centrilobular liver cells [10, 11]. The elevation of aminotransferase in cardiac disease is characterized by a greater increase in AST compared with ALT (due to the localization of these two enzymes). We hypothesized that the increase in AST superior to ALT is related to cardiovascular disease and it may be related to elevated BNP levels and subsequent cardiovascular mortality in health check-ups.

The purpose of the present study was to assess whether the ratio of AST to ALT (AST/ALT ratio) is associated with BNP levels and is a predictor for future cardiovascular mortality in the general population.

## 2. Methods

### 2.1. Ethics Statement and Study Population

The institutional ethics committee of Yamagata University School of Medicine approved the study, and all participants provided written informed consent. The procedures were performed in accordance with the Helsinki Declaration.

This study was a part of the ongoing Molecular Epidemiological Study, utilizing the resources of the Regional Characteristics of 21st Century Centers of Excellence (COE) Program and the Global COE in Japan, as previously described [12].

This study was based on a community-based annual health check-up of inhabitants from the town of Takahata in northern Japan (total population 26,026). Community members, aged > 40 years, were invited to participate. Between June 2004 and November 2007, 3,520 subjects (1,579 men and 1,941 women) were enrolled in the study. Subjects completed a self-reported questionnaire to document their medical history, current medication use, and clinical symptoms.

Twenty six subjects were excluded due to end-stage kidney dysfunction, incomplete data, or study withdrawal.

### 2.2. Measurement

Hypertension was defined as systolic blood pressure (BP) ≥ 140 mmHg, diastolic BP ≥ 90 mmHg, or antihypertensive medication use. Diabetes mellitus was defined as fasting blood sugar (FBS) ≥ 126 mg/dL, glycosylated hemoglobin A1c ≥ 6.5% (Japanese Diabetes Society value), or antidiabetic medication use.

### 2.3. Biochemical Markers

Blood samples for AST, ALT, and *γ*-glutamyl transpeptidase (*γ*-GTP) measurement were obtained early in the morning and the assay was performed according to the Japan Science of Clinical Chemistry (JSCC) recommendations. Blood samples were also obtained for measuring BNP. These samples were transferred to chilled tubes containing 4.5 mg ethylenediaminetetraacetic acid disodium salt and aprotinin (500 U/mL) and centrifuged at 1,000 ×g for 15 minutes at 4°C. The clarified plasma samples were frozen, stored at −70°C, and thawed just before the assay was performed. BNP concentrations were measured using a commercially available radioimmunoassay specific for human BNP (Shiono RIA BNP assay kit, Shionogi Co. Ltd., Tokyo, Japan) [13, 14].

Blood samples for measuring serum heart type fatty acid binding protein (H-FABP) concentrations were drawn and centrifuged at 2,500 ×g for 15 min at 4°C within 30 min of collection, and the obtained serum was stored at −70°C until analysis. H-FABP levels were measured using a two-step sandwich enzyme-linked immunosorbent assay (ELISA) kit (MARKIT-M H-FABP, Dainippon Pharmaceutical Co. Ltd., Tokyo, Japan), as previously described [15, 16].

Estimated glomerular filtration rate (eGFR) was calculated by the modification of diet in renal disease (MDRD) equation with the Japanese coefficient [17, 18].

### 2.4. Endpoint and Median Follow-Up in Years

All subjects were prospectively followed up for a median period of 9.3 years (interquartile range, 8.3–9.4 years). The endpoint was all-cause death, which was also broken down into cardiovascular deaths and noncardiovascular deaths.

### 2.5. Definition of High BNP and High AST/ALT Ratio

High BNP was defined as a BNP level ≥ 100 pg/mL, in accordance with previous reports and the Japanese heart failure guidelines [19, 20]. A high AST/ALT ratio was defined as >90 percentile of the study population.

### 2.6. Statistical Analysis

All values are expressed as the mean ± standard deviation. Continuous and categorical variables were compared with* t*-tests and chi-square tests, respectively. The Kruskal-Wallis test was used to compare the AST/ALT ratio with BNP levels. Univariate and multivariate logistic analyses were performed to determine the risk factors for a high BNP level. The receiver operating characteristics (ROC) curves for high BNP were constructed to calculate the area under the curve (AUC) for AST, ALT, and the AST/ALT ratio using the trapezoidal rule. A Cox proportional hazard analysis was performed to determine independent predictors for cardiovascular deaths, and significant predictors selected in the univariate analysis were entered into the multivariate analysis. The ROC curves for cardiovascular deaths were also illustrated and AUC was calculated to compare the prognostic capacities of biomarkers. Survival curves were constructed with the Kaplan-Meier method and compared using log-rank tests. A value of *P* < 0.05 was considered statistically significant. All statistical analyses were performed with a standard program package (JMP version 10, SAS Institute Inc., Cary, NC, USA).

## 3. Results

### 3.1. Comparison of Clinical Characteristics between Subjects with High and Low BNP Levels

The subject's baseline characteristics are shown in Table 1. There were 1,562 men and 1,932 women. The mean AST/ALT ratio was 1.18. Subjects were divided into two groups according to BNP level. Subjects with high BNP were older and had higher prevalence rates of male gender, previous cardiovascular disease, hypertension, and diabetes mellitus than those with low BNP. Also, subjects with a high BNP level had higher levels of systolic BP, H-FABP, and the AST/ALT ratio and lower levels of ALT and eGFR than those with low BNP. There were no significant differences in prevalence rate of previous cancer, previous liver disease, smoking, and alcohol consumption, HbA1c, FBS, AST, and *γ*-GTP between subjects with high and low BNP.

### 3.2. The Receiver Operating Characteristic Curve for High BNP

**Figure 1:**
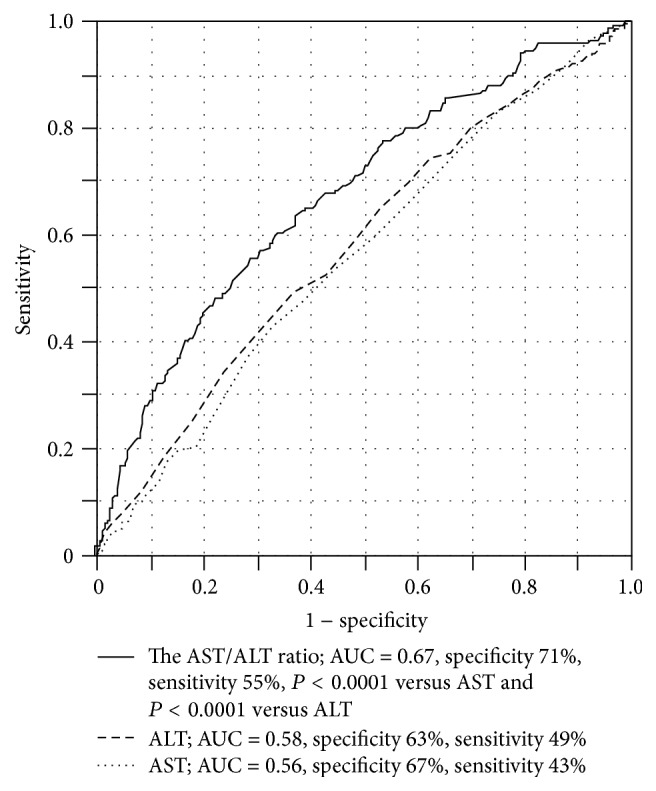
The receiver operating characteristics curve to predict high BNP levels. The area under the curve for the AST/ALT ratio was significantly greater than for AST and ALT. ALT, alanine transaminase; AST, aspartate transaminase; BNP, brain natriuretic peptide.

The ROC curves of AST, ALT, and AST/ALT ratio were illustrated to compare the predictive capacities of high BNP. The AUC, specificity, and sensitivity for the AST/ALT ratio were 0.67, 71%, and 55%, respectively. The AUC for the AST/ALT ratio was significantly greater than the AUC for AST and ALT, suggesting that the AST/ALT ratio improves the predictive capacity of high BNP (Figure 1).

### 3.3. Association between the AST/ALT Ratio and High BNP

**Figure 2:**
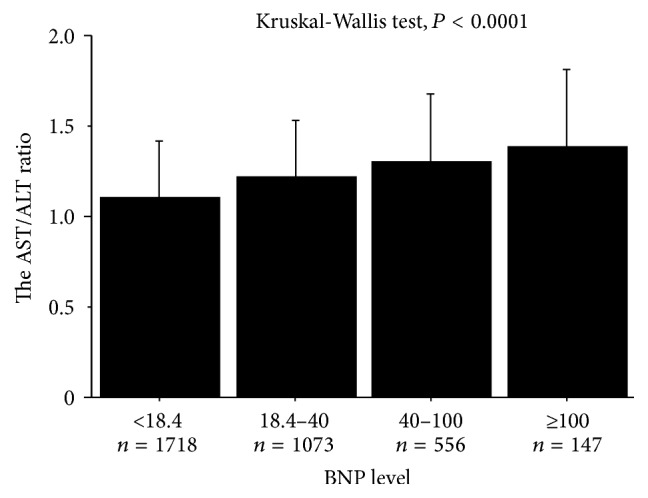
The association between the AST/ALT ratio and BNP level. The AST/ALT ratio was increased with increasing BNP levels (Kruskal-Wallis test, *P* < 0.0001). ALT, alanine transaminase; AST, aspartate transaminase; BNP, brain natriuretic peptide.

In the simple linear analysis, there was a significant correlation between the AST/ALT ratio and BNP (*r* = 0.277, *P* < 0.0001). As shown in Figure 2, the AST/ALT ratio was increased with increasing BNP level. To determine the risk factors for high BNP, univariate and multivariate logistic analyses were performed. In the univariate analysis, the AST/ALT ratio was significantly associated with high BNP (Table 2). In addition, age, male gender, previous cardiovascular disease, smoking, hypertension, diabetes mellitus, systolic BP, eGFR, and ALT were associated with high BNP. Multivariate logistic analysis showed that the AST/ALT ratio was significantly associated with high BNP after adjustment for age, male gender, previous cardiovascular disease, hypertension, diabetes mellitus, and eGFR (odds ratio, 1.31; 95% confidence interval, 1.13–1.53; *P* = 0.0004, Table 2).

### 3.4. The AST/ALT Ratio and Biomarkers

**Figure 3:**
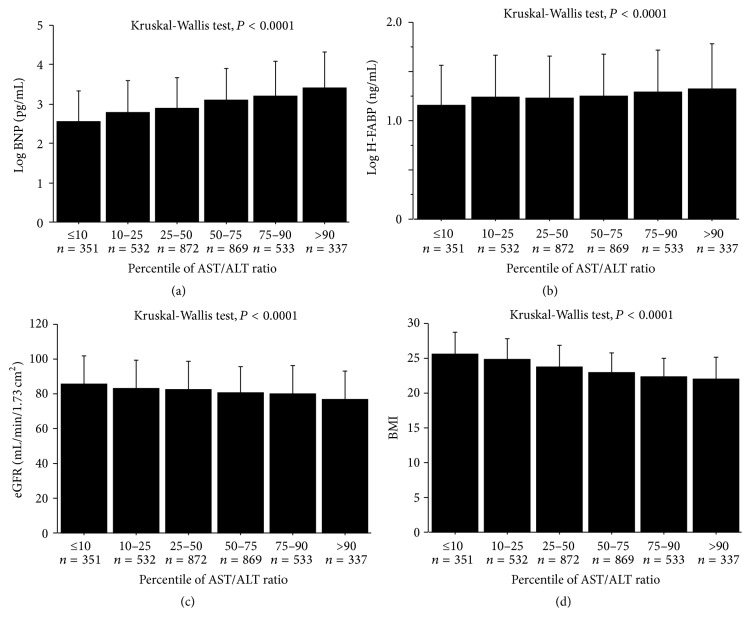
The association between the AST/ALT ratio and log BNP, log H-FABP, eGFR, and BMI. ALT, alanine transaminase; AST, aspartate transaminase; BMI, body mass index; BNP, brain natriuretic peptide; eGFR, estimated glomerular filtration rate; H-FABP, heart type fatty acid binding protein.

**Figure 4:**
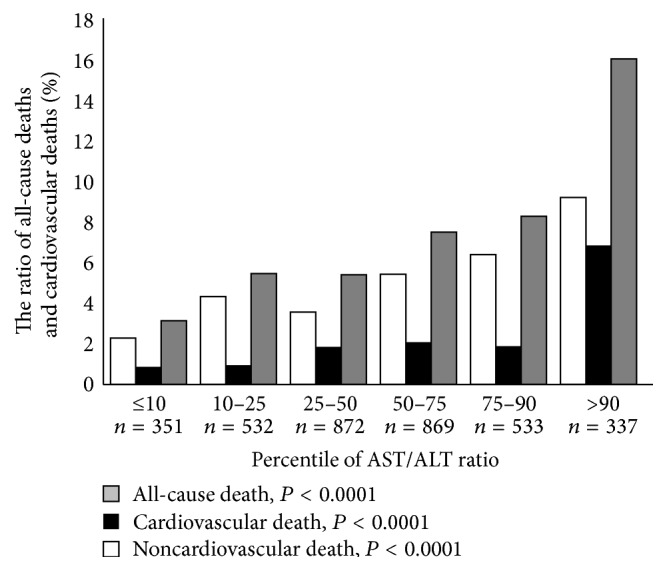
The association between the AST/ALT ratio and survival ratio. ALT, alanine transaminase; AST, aspartate transaminase.

To examine the association of the AST/ALT ratio with biomarkers, subjects were divided into 6 groups according to AST/ALT ratio percentiles: ≤10 percentile, *n* = 351; 10–25 percentile, *n* = 532; 25–50 percentile, *n* = 872; 50–75 percentile, *n* = 869; 75–90 percentile, *n* = 533; and >90 percentile, *n* = 337. As shown in Figures 3(a) and 3(b), cardiac biomarkers such as BNP and H-FABP were significantly increased with an increasing percentile of AST/ALT ratio. Furthermore, eGFR and body mass index (BMI) were significantly decreased with an increasing percentile of AST/ALT ratio. During the follow-up period, there were 250 all-cause deaths including 79 cardiovascular deaths. As shown in Figure 4, all-cause mortality, cardiovascular mortality, and noncardiovascular mortality were higher in subjects with a >90 percentile of AST/ALT ratio compared to other groups.

### 3.5. Cardiovascular Mortality and AST/ALT Ratio

To determine whether AST/ALT ratio can predict all-cause and cardiovascular mortality, we performed univariate and multivariate Cox proportional hazard regression analyses. In the univariate analysis, a high AST/ALT ratio was significantly associated with all-cause, cardiovascular, and noncardiovascular mortality (Table 3). A multivariate Cox proportional hazard regression analysis demonstrated that a high AST/ALT ratio was an independent predictor of future all-cause and cardiovascular mortality, but not noncardiovascular mortality, after adjusting for confounding risk factors (Table 3).

Next, we studied the statistical interaction between AST/ALT ratio and BNP using cut-off values of these biomarkers. Multivariate Cox proportional hazard regression analysis demonstrated that subjects with abnormal levels of AST/ALT ratio (≥1.6) and BNP (≥36.8 pg/mL) were at significantly increased risk for cardiovascular death after adjustments for abnormal AST/ALT ratio and abnormal BNP (hazard ratio, 4.18; 95% confidence interval, 1.14–16.39; *P* = 0.0328). To compare the prognostic capacity for cardiovascular deaths, ROC analyses were performed. As shown in Table 4, AUCs in BNP and AST/ALT ratio were 0.73 and 0.65, respectively. The AUC in AST/ALT ratio was greater than that in AST or ALT itself, indicating that transforming AST/ALT ratio improved the prognostic capacity compared to AST or ALT itself.

### 3.6. Comparisons of Clinical Characteristics of Subjects with a High and Low AST/ALT Ratio

**Figure 5:**
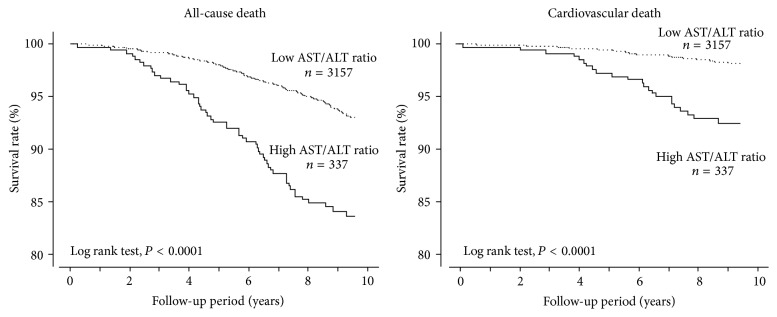
Kaplan-Meier analysis to predict all-cause and cardiovascular death between subjects with a high and low AST/ALT ratio. ALT, alanine transaminase; AST, aspartate transaminase.

As shown in Table 5, subjects with a high AST/ALT ratio were older and had higher prevalence rates of previous cardiovascular disease and lower prevalence rates of diabetes mellitus compared with those with a low AST/ALT ratio. Subjects with a high AST/ALT ratio also showed higher levels of BNP and H-FABP and lower levels of BMI, diastolic BP, HbA1c, FBS, and eGFR than those with a low AST/ALT ratio. There were no significant differences in gender, prevalence rate of previous cancer, previous liver disease, smoking, alcohol consumption, hypertension, and systolic BP between subjects with high and low AST/ALT ratios. Kaplan-Meier analysis demonstrated that both all-cause and cardiovascular mortality were higher in subjects with a high AST/ALT ratio than in subjects with a low AST/ALT ratio (Figure 5).

## 4. Discussion

The new and novel findings from this study were as follows: (1) the AST/ALT ratio was increased with increasing BNP levels; (2) multivariate logistic analysis demonstrated that a high AST/ALT ratio was significantly associated with a high BNP level; (3) multivariate Cox proportional hazard regression analysis demonstrated that the high AST/ALT ratio was an independent predictor of all-cause and cardiovascular mortality, but not noncardiovascular mortality; (4) subjects with a high AST/ALT ratio had higher levels of BNP and H-FABP and a lower level of eGFR; (5) Kaplan-Meier analysis demonstrated that all-cause and cardiovascular mortality were significantly higher in subjects with a high AST/ALT ratio than in those with a low AST/ALT ratio.

### 4.1. The AST/ALT Ratio Elevation in the General Population

The greater elevation of AST compared with ALT is reported to be due to induction by alcohol consumption and cardiohepatic interaction [5–7, 21, 22]. Although there were no significant differences in prevalence rates of alcohol consumption and *γ*-GTP levels, previous cardiovascular disease rate was higher in subjects with a high AST/ALT ratio than in those with a low AST/ALT ratio. These results suggest that an elevated AST/ALT ratio may reflect cardiac load and damage and suggest the presence of latent cardiovascular disease. Since AST is localized in myocardial tissue, a high AST/ALT ratio may represent AST leakage from the myocardial tissue secondary to myocardial damage. These findings indicated a close association of AST/ALT ratio with cardiovascular disease in the general population.

### 4.2. The AST/ALT Ratio and BNP Level

BNP levels are reported to be a useful biomarker for all-cause and cardiovascular mortality in the general population [3]. However, BNP levels are not routinely measured in health check-ups. On the other hand, laboratory assays for liver enzymes are common, readily available, and inexpensive. Since the AST/ALT ratio was correlated with BNP levels in the general population, it may be a significant biomarker for the identification of subjects with latent cardiovascular disease.

Serum BNP levels are affected by several factors such as BMI, kidney dysfunction, and cardiovascular disease [23, 24]. The inverse relationship between BMI and BNP is well known [23]. In addition, BNP levels are reported to increase with advancing kidney dysfunction [24]. Subjects with a high AST/ALT ratio had lower BMI and eGFR levels in the present study. These results supported our hypothesis that the AST/ALT ratio was closely associated with a high BNP level, which was associated with the presence of cardiovascular disease, low BMI, and kidney dysfunction.

### 4.3. The AST/ALT Ratio and Mortality

Recent studies focusing on liver enzymes demonstrated their prognostic capacity to predict cardiovascular and all-cause deaths in general populations. High aminotransferase activity is related to future mortality, whereas low ALT activity is also reported to be associated with future mortality in the general population [25–27]. Low ALT levels are reported to be associated with aging, frailty, and mortality in the elderly general population [28]. Decreased ALT is reported to result from a reduced liver size and liver blood flow [29]. In the present study, subjects with previous cardiovascular disease tended to have lower ALT levels, which may be associated with low liver blood flow as a result of latent myocardial damage. However, low ALT levels were not an independent predictor for high BNP levels. Thus, the prognostic utility of aminotransferase is limited due to its complex association with mortality in the general population. We demonstrated that the AST/ALT ratio predicts all-cause mortality and cardiovascular mortality. Since it was not significantly related to noncardiovascular mortality, the prognostic capacity of the AST/ALT ratio depended on its prediction of cardiovascular mortality. Considering the cardiohepatic interaction, the AST/ALT ratio could be a useful marker for cardiovascular mortality in the general population. We found a statistically significant interaction between AST/ALT ratio and BNP that predicted future cardiovascular deaths, indicating that the combination of AST/ALT ratio and BNP evaluations would be useful in determining risk in the general population. Therefore, clinical perspective of the present study was that AST/ALT ratio could be the screening tool for the high BNP subjects who are at high risk for cardiovascular disease and the additional information about cardiovascular mortality to BNP in general population.

## 5. Conclusions

The AST/ALT ratio was associated with abnormal BNP levels and cardiovascular mortality in the general population. Since laboratory assays for liver enzymes are common, readily available, and inexpensive, the AST/ALT ratio could be a promising parameter to identify subjects at high risk for cardiovascular disease and mortality in the general population.

## Figures and Tables

**Table 1:** Clinical characteristics of subjects with high and low BNP.

Variables	All subjects	Low BNP	High BNP	*P* value
	*n* = 3494	*n* = 3347	*n* = 147	
Age (years)	62 ± 10	62 ± 10	72 ± 8	<0.0001
Men/women	1562/1932	1484/1863	78/69	0.0373
Previous CVD, *n* (%)	459 (13%)	394 (12%)	65 (44%)	<0.0001
Previous cancer, *n* (%)	74 (2.1%)	70 (2.1%)	4 (2.7%)	0.6038
Previous liver disease, *n* (%)	82 (2.3%)	80 (2.4%)	2 (1.4%)	0.4196
Smoking, *n* (%)	1121 (32%)	1065 (32%)	56 (38%)	0.1106
Alcohol consumption, *n* (%)	1454 (42%)	1390 (42%)	64 (44%)	0.6289
Hypertension, *n* (%)	1278 (37%)	1196 (36%)	82 (56%)	<0.0001
Diabetes mellitus, *n* (%)	241 (7%)	224 (7%)	17 (12%)	0.0225
Systolic BP, mmHg	134 ± 16	134 ± 16	138 ± 17	0.0016
Diastolic BP, mmHg	80 ± 10	80 ± 10	80 ± 10	0.6310
HbA1c, %	5.7 ± 0.7	5.7 ± 0.7	5.7 ± 0.7	0.1783
FBS, mg/dL	95 ± 17	95 ± 17	96 ± 15	0.6199
eGFR, mL/min/1.73 m^2^	82 ± 16	82 ± 16	71 ± 19	<0.0001
Log H-FABP, ng/mL	1.24 ± 0.43	1.23 ± 0.43	1.56 ± 0.39	<0.0001
AST, IU/L	24.6 ± 8.3	24.9 ± 12.3	26.2 ± 9.6	0.2329
ALT, IU/L	22.9 ± 11.6	23.5 ± 14.2	20.9 ± 11.3	0.0273
*γ*-GTP, IU/L	35 ± 42	36 ± 47	39 ± 52	0.3517
AST/ALT ratio	1.18 ± 0.34	1.17 ± 0.34	1.38 ± 0.43	<0.0001

Data are expressed as mean ± standard deviation or number (%).

ALT, alanine transaminase; AST, aspartate transaminase; BNP, brain natriuretic peptide; BP, blood pressure; CVD, cardiovascular disease; eGFR, estimated glomerular filtration rate; FBS, fasting blood sugar; *γ*-GTP, gamma glutamyl transpeptidase; HbA1c, glycosylated hemoglobin A1c; H-FABP, heart type fatty acid binding protein.

**Table 2:** Univariate and multivariate logistic analyses to predict high BNP.

Variables	Univariate analysis			Multivariate analysis		
	OR	95% CI	*P* value	OR	95% CI	*P* value
Age (years)	1.12	1.10–1.14	<0.0001	1.08	1.05–1.10	<0.0001
Men/women	0.71	0.51–0.98	0.0382	0.76	0.54–1.08	0.1300
BMI	0.97	0.92–1.02	0.2485			
Previous CVD	5.94	4.22–8.36	<0.0001	3.53	2.46–5.07	<0.0001
Previous cancer	1.31	0.47–3.64	0.6049			
Previous liver disease	0.56	0.14–2.31	0.4259			
Smoking	1.32	0.94–1.85	0.1116			
Alcohol consumption	1.09	0.77–1.52	0.6289			
Hypertension	2.27	1.63–3.12	<0.0001	1.18	0.82–1.70	0.3697
Diabetes mellitus	1.82	1.08–3.08	0.0245	1.55	0.88–2.72	0.1258
Systolic BP	1.02	1.01–1.03	0.0016			
Diastolic BP	1.00	0.99–1.02	0.6309			
HbA1c^*∗*^	1.10	0.96–1.27	0.1785			
FBS^*∗*^	1.03	0.84–1.18	0.6466			
eGFR^*∗*^	0.47	0.37–0.57	<0.0001	0.71	0.58–0.86	0.0007
AST^*∗*^	1.04	0.97–1.12	0.2523			
ALT^*∗*^	0.79	0.65–0.97	0.0234			
*γ*-GTP^*∗*^	1.04	0.66–1.18	0.3552			
AST/ALT ratio^*∗*^	1.59	1.39–1.81	<0.0001	1.31	1.13–1.53	0.0004

ALT, alanine transaminase; AST, aspartate transaminase; BMI, body mass index; BNP, brain natriuretic peptide; BP, blood pressure; CVD, cardiovascular disease; eGFR, estimated glomerular filtration rate; FBS, fasting blood sugar; *γ*-GTP, gamma glutamyl transpeptidase; HbA1c, glycosylated hemoglobin A1c; OR, odds ratio.

^*∗*^Per 1-SD increase.

**Table 3:** Univariate and multivariate Cox proportional hazard regression analyses for all-cause, cardiovascular, and noncardiovascular mortality.

Variables	Unadjusted			Adjusted		
	HR	95% CI	*P* value	HR	95% CI	*P* value
All-cause mortality						
High AST/ALT ratio	2.70	2.00–3.65	<0.0001	1.46^*∗*^	1.06–2.00	0.0190
				1.43^#^	1.04–1.96	0.0284
Cardiovascular mortality						
High AST/ALT ratio	4.35	2.65–7.09	<0.0001	2.19^*∗*^	1.30–3.69	0.0031
				2.51^#^	1.49–4.24	0.0006
Noncardiovascular mortality						
High AST/ALT ratio	2.12	1.44–3.13	0.0001	1.18^*∗*^	0.79–1.77	0.4109
				1.12^#^	0.75–1.69	0.5800

^*∗*^Adjusted for age, gender, consumption of alcohol, smoking, hypertension, diabetes mellitus, and eGFR.

^#^Adjusted for age, gender, body mass index, previous cardiovascular disease, diabetes mellitus, eGFR, and BNP.

ALT, alanine transaminase; AST, aspartate transaminase; CI, confidence interval; eGFR, estimated glomerular filtration rate; HR, hazard ratio.

**Table 4:** The area under the curves for cardiovascular deaths in general population.

	Area under the curve
BNP	0.73
eGFR	0.64
*γ*-GTP	0.55
AST	0.56
ALT	0.56
AST/ALT ratio	0.65

ALT, alanine transaminase; AST, aspartate transaminase; BNP, brain natriuretic peptide; eGFR, estimated glomerular filtration rate; *γ*-GTP, gamma glutamyl transpeptidase.

**Table 5:** Clinical characteristics between subjects with high and low AST/ALT ratio.

Variables	Low AST/ALT ratio	High AST/ALT ratio	*P* value
	*n* = 3157	*n* = 337	
Age (years)	62 ± 10	68 ± 11	<0.0001
Men/women	160/177	1402/1755	0.2815
BMI, kg/cm^2^	23.7 ± 3.1	22.1 ± 3.1	<0.0001
Previous CVD, *n* (%)	380 (12%)	79 (22%)	<0.0001
Previous cancer, *n* (%)	62 (2%)	12 (3%)	0.0869
Previous liver disease, *n* (%)	73 (2%)	9 (3%)	0.8255
Smoking, *n* (%)	998 (32%)	123 (34%)	0.3306
Alcohol consumption, *n* (%)	1291 (41%)	163 (46%)	0.1125
Hypertension, *n* (%)	1134 (36%)	144 (40%)	0.1305
Diabetes mellitus, *n* (%)	229 (7%)	12 (3%)	0.0052
Systolic BP, mmHg	134 ± 16	135 ± 17	0.4902
Diastolic BP, mmHg	80 ± 10	78 ± 10	0.0050
HbA1c, %	5.7 ± 0.7	5.5 ± 0.5	<0.0001
FBS, mg/dL	95 ± 17	92 ± 12	0.0004
eGFR, mL/min/1.73 m^2^	82 ± 16	77 ± 16	<0.0001
Log H-FABP, ng/mL	1.23 ± 0.43	1.34 ± 0.45	<0.0001
Log BNP, pg/mL	2.95 ± 0.84	3.42 ± 0.90	<0.0001
AST, IU/L	24.8 ± 9.7	27.4 ± 25	0.0002
ALT, IU/L	24.3 ± 13.8	15.0 ± 13.2	<0.0001
*γ*-GTP, IU/L	36 ± 45	34 ± 66	0.3455

Data are expressed as mean ± standard deviation or number (%).

ALT, alanine transaminase; AST, aspartate transaminase; BMI, body mass index; BNP, brain natriuretic peptide; BP, blood pressure; CVD, cardiovascular disease; eGFR, estimated glomerular filtration rate; FBS, fasting blood sugar; *γ*-GTP, gamma glutamyl transpeptidase; HbA1c, glycosylated hemoglobin A1c; H-FABP, heart type fatty acid binding protein.
